# Whole-genome sequencing in Brazilian patients with neurofibromatosis type 1, including novel variants, incidental findings, and dual diagnoses

**DOI:** 10.31744/einstein_journal/2026AO2160

**Published:** 2026-06-23

**Authors:** Luise Longo Angeloni, Josep Jorente, Ruy Pires de Oliveira, Vera Lúcia Gil-da-Silva-Lopes, Carolina Gama Vidoti-Nascimento, Mara Sanches Guaragna, Tarsis Paiva Vieira, Carlos Eduardo Steiner, The Brazilian Rare Genomes Project Consortium, Anne Caroline Barbosa Teixeira, Antonio Victor Campos Coelho, Caio Robledo D’ Angioli Costa Quaio, Carolina Araujo Moreno, Eduardo Perrone, Jose Bandeira do Nascimento, Jessica Grasiela Araujo Espolaor, Joana Rosa Marques Prota, Joao Bosco de Oliveira, Jose Ricardo Magliocco Ceroni, Kelin Chen, Letícia Torres Ferreira, Lucas Santos de Santana, Luciana Souto Mofatto, Luiza do Amaral Virmond, Marina de Franca Basto Silva, Michele Patricia Migliavacca, Renata Moldenhauer Minillo, Renata Yoshiko Yamada, Roberta Sitnik, Tatiana Ferreira de Almeida, Thiago Yoshinaga Tonholo Silva, Vivian Pedigone Cintra

**Affiliations:** 1 Universidade Estadual de Campinas Faculdade de Ciências Médicas Department of Medical Genetics and Genomic Medicine Campinas SP Brazil Department of Medical Genetics and Genomic Medicine, Faculdade de Ciências Médicas, Universidade Estadual de Campinas, Campinas, SP, Brazil.; 2 Hospital Israelita Albert Einstein São Paulo SP Brazil Hospital Israelita Albert Einstein, São Paulo, SP, Brazil

**Keywords:** Neurofibromatosis 1, Neurofibromatosis-noonan, Whole genome sequencing, Optical genome mapping, Incidental findings, Double diagnosis, DNAH5, KCND3

## Abstract

**Objective:**

To describe clinical and molecular aspects of a cohort of Brazilian individuals with neurofibromatosis type 1, a neurocutaneous disorder associated with a predisposition to tumors and inter- and intrafamilial variable expressivity.

**Methods:**

We conducted a retrospective study of 50 patients from 30 unrelated families, with features of Neurofibromatosis type 1, who underwent clinical evaluation and whole genome sequencing.

**Results:**

Patient ages ranged from 9 months to 62 years (mean 21.7 years). The most frequent manifestations were café-au-lait (100%), lentiginous macules (94%), Lisch nodules (62%), cutaneous (62%), and plexiform (38%) neurofibromas. Other findings included neurodevelopmental disorders (14%), congenital pseudarthrosis of the tibia (4%), optic pathway glioma (4%), and sphenoid wing dysplasia (2%). Eight individuals presented with tumors other than neurofibromas, including two with malignant peripheral nerve sheath tumors, two with breast cancer, and one each with a dysembryoplastic neuroepithelial tumor, cholangiocarcinoma, pilocytic astrocytoma, and basal cell carcinoma. All probands, except one, tested positive for pathogenic or likely pathogenic variants, of which three (11.1%) were novel (c.3372del, c.7299del, and c.7457_7457+2del), three were recurrent within this series (c.3826C>T, c.5902C>T, and c.6855C>A), and the others were private. Two families (6.6%) presented incidental findings, one in *BRCA1* and the other in *TMEM127*. Three individuals (6.5%) had a second molecular diagnosis: one each with spinocerebellar ataxia type 19, primary ciliary dyskinesia type 3, and XYY syndrome.

**Conclusion:**

This study presents the largest cohort of Brazilian individuals with neurofibromatosis type 1 to utilize whole-genome sequencing, identifying three novel germline *NF1* variants and two previously unreported double diagnoses.

## In Brief

Angeloni et al. contributed genomic data from a cohort of Brazilian individuals with Neurofibromatosis type 1, describing the clinical and molecular features of 50 patients from 30 unrelated families who underwent whole-genome sequencing. Novel variants in the NF1 gene are reported. Incidental findings and rare dual-molecular diagnoses have also been presented.

## Highlights

■ This is the largest cohort of individuals with NF1 investigated through whole-genome sequencing.
■ Molecular data contributed to the representation of the Brazilian population in genomic research.
■ Novel variants in the NF1 gene have been described, including c.3372del, c.7299del, and c.7457_7457+2del.
■ The first report of spinocerebellar ataxia type 19 in Brazil is also presented.

## Graphical abstract

**Figure:**
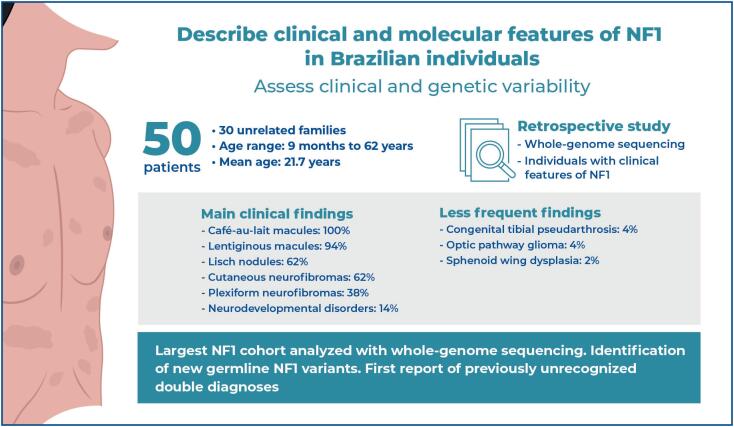

## INTRODUCTION

Neurofibromatosis type 1 (NF1; OMIM #162200) is a neurocutaneous disorder with autosomal dominant inheritance caused by germline mutations in the *NF1* gene on chromosome 17q11.2. It is associated with a somatic event ("second hit") leading to a loss of heterozygosity, a molecular physiopathological event at the cellular level.^(1,2)^ As part of the RAS/MAPK pathway, the *NF1* gene encodes neurofibromin, a large GTPase-activating protein that negatively regulates Ras signaling. Loss of neurofibromin expression, commonly observed in NF1-associated tumors, results in elevated levels of active Ras, leading to enhanced cell growth and survival through hyperactivation of the Ras pathway.^(3)^ Its large size may explain why *NF1* has one of the highest known mutation rates among humans, leading to a disease frequency of approximately 1 in 2,000-3,500 individuals, with approximately 50% of cases resulting from *de novo* events. ^(4,5)^

Neurofibromatosis type 1 is the most common genetic disease with a predisposition to malignancies^(3,6)^ and one of the most well-recognized genetic disorders, documented in pre-modern medicine even before its official description as a clinical entity at the end of the 19th century. ^(7)^

The nomenclature and diagnostic criteria have been standardized since 1987, and the most recent consensus was reached in 2021. NF1 is characterized by a combination of pigmentary cutaneous changes, mostly multiple café-au-lait macules (CALMs) and lentiginous macules (LMs) in non-sun-exposed areas; ocular manifestations, such as iris hamartomas (Lisch nodules, LNs) and choroidal abnormalities; and skeletal defects, including sphenoid wing dysplasia, anterolateral bowing or congenital pseudarthrosis of the tibia (CPT), or pseudarthrosis of another long bone. In addition, there is a predisposition to tumors, the most common of which are neurofibromas, including cutaneous (cNF) or plexiform (pNF), optic pathway gliomas, malignant peripheral nerve sheath tumors (MPNST), breast cancer, and brain tumors.^(1,8)^ It presents complete penetrance after adolescence and highly variable expressivity, even within affected members of the same family, and nearly all features are age-dependent. Thus, in nonfamilial cases, clinical diagnosis can be challenging at a young age if only isolated CALMs are present.^(2, 4, 9, 10)^

Diagnosis can also be established by detecting a heterozygous pathogenic *NF1* variant with an allele fraction of 50% in apparently normal tissues, such as white blood cells.^(1)^ Traditional next-generation sequencing techniques can identify causative variants in 95-97% of cases. Except for low-sensitivity technical failures with low coverage, the remaining clinically diagnosed individuals may present with mosaicism or structural chromosomal rearrangements that interrupt the *NF1* sequence, including translocations (particularly the recurrent 17;22) or complex intragenic recombinations. In such cases, optical genome mapping (OGM) is an alternative to molecular investigations. ^(2)^

The Brazilian Rare Genomes Project (BRGP) is a public/private initiative that incorporates whole-genome sequencing (WGS) to enhance the diagnosis of rare genetic diseases and to integrate genomic precision medicine into the Brazilian public healthcare system. Early adoption of WGS for the investigation of rare diseases can be beneficial, as it shortens the diagnostic odyssey and yields a diagnostic rate higher than that of other next-generation techniques such as whole exome and targeted or panel sequencing. Moreover, WGS enables the detection of mitochondrial, copy number, and structural variants, as well as the analysis of non-coding regions. In some cases, depending on the reading technology, expansion repeat variants may also be detected. Thus, this reduces the time and cost associated with multiple sequential genetic tests and provides a cost-effective genomic testing strategy.^(11)^ The BRGP conducts molecular diagnoses using WGS for patients with rare diseases or hereditary cancer who are undergoing clinical investigation and/or follow-up at 21 of the 32 Reference Centers for Rare Diseases nationwide.

## OBJECTIVE

This study aimed to contribute to the understanding of neurofibromatosis in Brazil by providing clinical and molecular data, ultimately enhancing knowledge of the disorder's genotypic and phenotypic variations in the Brazilian population.

## METHODS

This retrospective case series included individuals diagnosed with NF1 at a clinical genetics service within a Reference Center for Rare Diseases participating in the BRGP. The study population comprised probands who underwent WGS and their family members. Collected data encompassed sex, age at the first hospital appointment, clinical features, complementary findings, family history, and molecular results. This study was approved by the institutional ethics committee, and all participants provided informed consent prior to data and blood sample collection.

Molecular analyses were performed according to the BRGP protocol. The WGS workflow consisted of three major steps: wet laboratory sample processing, bioinformatics analyses for variant calling and annotation, and correlation of clinical and molecular findings, resulting in a medical report. Detailed technical information on every step can be obtained from Coelho et al.^(11)^ In summary, DNA was extracted from whole blood samples using the QIAsymphony DNA Mini Kit on the QIAsymphony automated system (both Qiagen, Valencia, CA, USA) and subjected to WGS on an Illumina platform. Data were processed to detect sequence variants, copy number variations, and structural variants in accordance with the best practices for the bioinformatics pipeline. Quality metrics included a minimum coverage of 20× and at least 90% coverage at depths above 15×. The reference genome used was GRCh38/hg38.^(11)^ Variants’ nomenclature and classification followed the ACMG recommendations^(12,13)^ with refinements proposed by the standards for constitutional sequence variant classification, adapted to the specific characteristics of the Brazilian population.^(14)^ These analyses were performed using the VarStation platform version 3.0. The results were compared with available data from the PubMed and SciELO databases. Variants were considered novel when they were absent from the literature, ClinVar, LOVD, ABRAOM, 3Billion, and HGMD databases.

Optical genome mapping was performed on DNA extracted from peripheral blood samples at the Uniscience Molecular Laboratory, São Paulo, Brazil. High-molecular-weight DNA was extracted using the Bionano Prep Blood and Cell Culture DNA Isolation kit, according to the manufacturer's instructions. Briefly, high-molecular-weight DNA was labeled using the Bionano Prep direct label and stain (DLS) method and loaded into a flow cell for analysis using the Saphyr Optical Mapping system. The raw molecule data obtained via optical mapping were analyzed using a bioinformatics pipeline to remove molecules of less than 150 kb and fewer than nine motifs per molecule for *de novo* genome assembly. Finally, these data were aligned to an *in silico* reference genome (GRCh38) using the Bionano Solve v3.5 RefAligner module. Structural variants were identified by comparing the genome with the reference genome using a customized Bionano SV caller. The results were analyzed using the GRCh38 reference genome and the Bionano Access software program v1.5.1.

### Institutional review board statement

This study was conducted in accordance with the Declaration of Helsinki and approved by the Institutional Ethics Committee Board (*Universidade Estadual de Campinas*) CAAE: 29567220.4.2005.5404; 4.495.622.

### Informed consent statement

All patients or their parents/guardians were informed prior to data collection, and written informed consent was obtained for the publication of their medical cases and accompanying images.

## RESULTS

**Figure 1:**
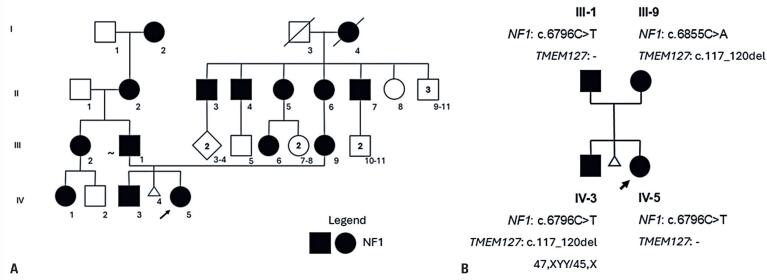
Pedigree of family 2 (A) and partial pedigree with genomic data of the investigated individuals (B)

Between April 2004 and December 2021, 134 patients were clinically diagnosed with NF1 at the Clinical Genetics Service of the *Universidade Estadual de Campinas* Teaching Hospital. For this study, only individuals undergoing periodic follow-up between 2021 and 2023 were invited to join the BRGP recruitment phase. Following the invitation and consent procedures, 30 probands and their relatives agreed to participate and signed the consent form, forming a cohort subset of 50 individuals from 30 unrelated families (sex ratio of 20 males to 30 females). The age at first hospital appointment ranged from 9 months to 62 years (mean: 21.7, median: 17 years). The primary reasons for referral to a clinical geneticist were to confirm NF1 suspicion or provide specialized follow-up when the diagnosis had already been established. Family history was positive in 11/30 (36.6%) probands, negative in 18/30 (60%), and unavailable in 1 case (3.3%). Family 2 presented with a biparental history (Figure 1). Consanguinity was identified in one proband (Patient 29), born to a second-cousin-once-removed couple.

**Figure 2:**
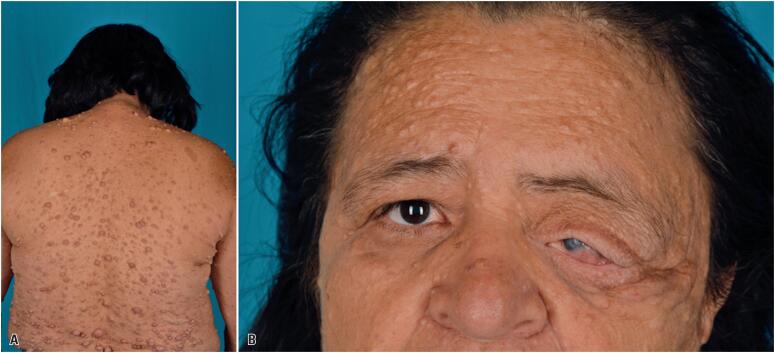
Patient 25-M showing café-au-lait macules and multiple cutaneous neurofibromas (A), and facial asymmetry due to sphenoid wing dysplasia and optic pathway glioma of the left eye (B)

In the general cohort, CALMs were present in 50/50 (100%) individuals, LMs in 47/50 (94%), cNF in 31/50 (62%), and pNF in 19/50 (38%). Among those who underwent targeted examinations, LNs were identified in 31/44 (62%). Optic pathway gliomas were found in two individuals (4%), sphenoid wing dysplasia in one (Patient 25-Mother, Figure 2), and congenital pseudarthrosis of the tibia in two (4%). Beyond these core findings, neurodevelopmental disorders (NDD), including attention deficit hyperactivity disorder, learning disability, and/or developmental delay, were present in seven individuals (14%). Atypical symptoms occurred in two cases: Patient 9, who developed progressive cerebellar ataxia at age 47 years (characterized by gait abnormalities, dysarthria, dysphagia, and cerebellar atrophy), whereas Patient 30 presented with *situs inversus totalis* and recurrent bronchopulmonary infections. Clinical findings for all 50 individuals are summarized in Table 1S, Supplementary Material.

Eight individuals presented with tumors other than cNF or pNF, including one case each of dysembryoplastic neuroepithelial tumor (Patient 3), cholangiocarcinoma (Patient 5-M), unilateral invasive ductal carcinoma (Patient 9), MPNST of the right thigh (Patient 10-M), basal cell carcinoma (Patient 11-M), estrogen/progesterone receptor-positive invasive carcinoma (Patient 16), MPNST of the dorsum (Patient 18), and pilocytic astrocytoma (Patient 26).

**Figure 3:**
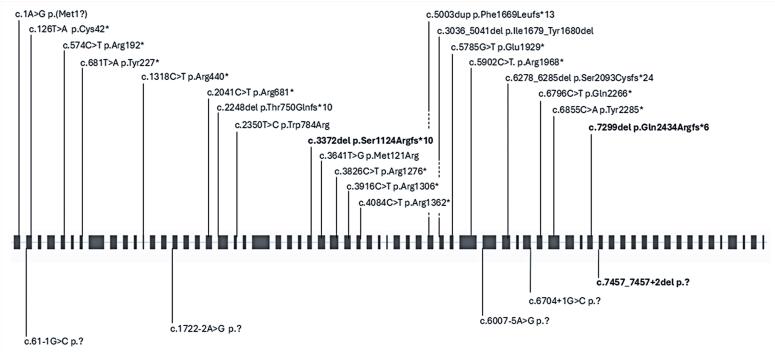
Schematic representation of the *NF1* gene on chromosome 17q11.2 (transcript NM_001042492.3, genome build GRCh38/hg38) with the location of the variants detected in this cohort. Black boxes represent exons, whereas continuous lines represent introns. In the upper part of the figure, the exonic variants are shown, and in the lower part, the canonical splice site variants are depicted; the novel variants are contrasted in bold

Regarding molecular results, individual 1 was the only case without structural or sequence variants identified via WGS and OGM analyses. Conversely, in biparental family 2, two variants were identified, yielding a total of 30 positive results. Figure 3 illustrates the distribution of variants along the *NF1* gene.

All probands carried pathogenic or likely pathogenic *NF1* variants, with three recurrently observed within the cohort: c.3826C>T p.Arg1276* in patients 7 and 25, c.5902C>T p.(Arg1968*) in patients 13 and 22, and c.6855C>A p.(Tyr2285*) in patients 2-M and 16. The remaining variants were private to each family, including 3/27 (11.1%) novel variants. Nonsense variants were the most frequent type (13/27; 48.1%), followed by frameshift and intronic variants (5/27; 18.5% each), and missense variants (2/27; 7.4%). In-frame deletions, start-loss, and gene deletion were observed in one individual each (3.7%). Table 1 summarizes the WGS variants identified via WGS analysis.

**Table 1:** Clinical synopsis and variants in the *NF1* gene that were identified in each proband, along with their classification according to ACMG criteria

Proband	Clinical synopsis	Variant	Type ACMG Classification	References
1	CALMs, LMs, ADHD	-	-	-
2	CALMs, LMs, LNs	c.6796C>T p.(Gln2266*)	nonsense pathogenic	ClinVar ID 545966
2-M	CALMs, LMs, cNF	c.6855C>A p.(Tyr2285*)	nonsense pathogenic	Scala et al. ^(6)^
3	CALMs, LMs, cNF, pNF, LNs, dysembryoplastic neuroepithelial tumor	c.2350T>C p.Trp784Arg	missense pathogenic	Kluwe et al. ^(15)^
4	CALMs, LMs, cNF, LNs, LD, mild ID, congenital hip dislocation	del(17)(q11.2q11.2)	Copy Number Variation pathogenic	Kayes et al. ^(16)^
5	CALMs, LMs, cNF, LNs, CPT	c.5785G>T p.(Glu1929*)	nonsense pathogenic	ClinVar ID 852822
6	CALMs, LMs, cNF	c.4084C>T p.(Arg1362*)	nonsense pathogenic	Upadhyaya et al. ^(17)^
7	CALMs, LMs, cNF, pNF, LNs	c.3826C>T p.Arg1276*	nonsense pathogenic	Heim et al. ^(18)^
8	CALMs, LMs, LNs	c.3372del p.(Ser1124Argfs*18)	frameshift pathogenic	novel
9	CALMs, LMs, cNF, pNF, LNs, breast cancer, cerebellar ataxia	c.1722-2A>G p.?	canonical splice site pathogenic	Wimmer et al. ^(19)^
10	CALMs, LMs, cNF, LNs	c.574C>T p.Arg192*	nonsense pathogenic	Fahsold et al. ^(20)^ Toliat et al. ^(21)^ Messiaen et al. ^(22)^
11	CALMs, LMs, cNF, LNs	c.7457_7457+2del p.?	canonical splice site pathogenic	novel
12	CALMs, LMs, cNF, pNF	c.5003dup p.(Phe1669Leufs*13)	frameshift pathogenic	LOVD ID #0000219563
13	CALMs, LMs, cNF, pNF, LNs	c.5902C>T p.(Arg1968*)	nonsense pathogenic	Cawthon et al. ^(23)^
14	CALMs, LMs	c.6007-5A>G p.?	splice region pathogenic	Ainsworth et al. ^(24)^
15	CALMs, LMs, LNs	c.7299del p.(Gln2434Argfs*6)	frameshift pathogenic	novel
16	CALMs, LMs, cNF, pNF, breast cancer	c.6855C>A p.(Tyr2285*)	nonsense pathogenic	Scala et al. ^(6)^
17	CALMs, LMs, cNF, pNF, LNs	c.5036_5041del p.(Ile1679_Tyr1680del)	in-frame deletion likely-pathogenic	Wu et al. ^(25)^
18	CALMs, LMs, cNF, pNF, LNs, MPNST, mild ID	c.1318C>T p.(Arg440*)	nonsense pathogenic	Heim et al. ^(18)^
19	CALMs, LMs, LNs	c.2248del p.(Thr750Glnfs*10)	frameshift pathogenic	3Billion SCV002012239.1 (ID 1320213)
20	CALMs, LMs, Neurofibromatosis-Noonan phenotype	c.1A>G p.(Met1?)	start loss likely-pathogenic	Ko et al. ^(26)^
21	CALMs, LMs	c.2041C>T p.Arg681*	nonsense pathogenic	Ars et al. ^(27)^
22	CALMs, LMs, cNF, LNs	c.5902C>T p.(Arg1968*)	nonsense pathogenic	Cawthon et al. ^(23)^
23	CALMs, LMs, cNF, pNF, LNs	c.61-1G>C p.?	canonical splice site likely-pathogenic	ClinVar ID 3338872
24	CALMs, LMs, cNF, pNF, LNs	c.126T>A p.(Cys42*)	nonsense pathogenic	ClinVar ID 2768055
25	CALMs, LMs, cNF	c.3826C>T p.Arg1276*	nonsense pathogenic	Heim et al. ^(18)^
26	CALMs, LMs, cNF, LNs, pilocytic astrocytoma	c.681T>A p.(Tyr227*)	nonsense pathogenic	ClinVar ID 580256
27	CALMs, DD, macrocrania	c.3641T>G p.(Met1214Arg)	missense likely-pathogenic	ClinVar ID 570979
28	CALMs, LMs	c.6704+1G>C p.?	canonical splice site pathogenic	Nemethova et al. ^(28)^
29	CALMs, LMs, CPT, DD	c.3916C>T p.Arg1306*	nonsense pathogenic	Fahsold et al. ^(20)^
30	CALMs, LMs, *situs inversus totalis*, recurrent pulmonary infections	c.6278_6285del p.(Ser2093Cysfs*24)	frameshift pathogenic	ClinVar ID 2737151

Note: Transcript NM_001042492.3, reference genome GRCh38 - hg38; all variants in heterozygosity.

ADHD: attention deficit hyperactivity disorder; cNF: cutaneous neurofibroma; CPT: congenital pseudarthrosis of the tibia; DD: developmental delay; CALM: café-au-lait macules; ID: intellectual deficiency; LD: learning disability; LMs: lentiginous macules; LNs: lisch nodules; MPNST: malignant peripheral nerve sheath tumor; pNF: plexiform neurofibroma(s).

Beyond the primary *NF1* mutation, two families exhibited additional molecular findings: one family harbored a heterozygous pathogenic *BRCA1* variant (c.5074+2T>C p.?) (patients 5 and 5-M), whereas another carried a heterozygous pathogenic *TMEM127* variant (c.117_120del; p.(Ile41Argfs*39)) (patients 2-B and 2-M). Three individuals presented with a second molecular variant of uncertain significance (VUS): patient 9 with the heterozygous *KCND3* variant (c.1496C>G p.(Ser499Cys)), and patient 30 with heterozygous *DNAH5* variants (c.8311C>T p.(Arg2771Cys) and c.8010+3A>G p.?). Furthermore, Patient 2-B was found to have a duplicated Y chromosome, with cytogenetic analysis revealing a 47,XYY[44]/45,X[6] constitution.

## DISCUSSION

Brazil exhibits the most pronounced genomic diversity worldwide; however, its population remains underrepresented in genomic research.^(29)^ The present study addresses this gap by analyzing genomic data from a cohort of Brazilian individuals with NF1.

While most NF1 studies are pilot-tested in adult or pediatric oncology samples, this study was conducted within a single clinical genetics service. Rather than a population-based study, this was a convenience sample of individuals seen at a reference center for rare diseases. The sex ratio and family data were similar to a study of 101 Brazilian adults with NF1, which focused on sociodemographic factors and the impact of the disease on quality of life.^(30)^ Participants in the present study were referred for diagnostic confirmation and/or clinical follow-up. Consequently, this series cannot determine the true age at diagnosis, as several individuals had already received a clinical diagnosis prior to referral. However, the age at the first hospital appointment indicates a referral delay, likely reflecting low disease awareness among generalists and other medical specialists. Furthermore, because all individuals in this group met at least two evidence-based diagnostic criteria for NF1 via simple physical examination, and nearly 40% of probands had a positive family history, prompt clinical suspicion and referral should have been possible.

The most frequent clinical findings in this series were CALMs (100%) and LMs (94%). However, LMs were present in all individuals over the age of five, reflecting, along with cNF, the age-dependent nature of this disorder. Most patients presented with uncomplicated disease manifestations characterized by pigmentary changes and cNF alone; none were reported to have dystrophic scoliosis or early-onset osteoporosis. The sample size and young age of many participants may explain these findings.

While the 14% rate of neurodevelopmental disorders in this cohort, usually mild, exceeded that of the general population, it was lower than the rate of learning difficulties reported by van Minkelen et al.^(31)^ in a large Dutch NF1 cohort. In the present study, only one individual (3.3%; patient 4) exhibited a microdeletion of the entire *NF1* gene. Such microdeletions are associated with a six-fold higher risk for special education needs compared to intragenic variants in the *NF1* gene^(31)^ and are expected to occur in nearly 10% of cases.^(5,9)^

Regarding family history, family 2 was of particular interest because both parents and both children were affected; the couple also had a spontaneous first-trimester miscarriage, which was not further investigated. The possibility of biparental inheritance in this family was tested, assuming that the homozygosity of germline *NF1* variants would be lethal because of early embryonic mortality. Nevertheless, Alghamdi et al.^(32)^ described a 3-month-old boy with CALMs and juvenile myelomonocytic leukemia carrying a pathogenic *NF1* variant (c.586+5G>A) in a mosaic homozygous state, with higher frequency in blood DNA reads and lower frequency in saliva- and skin-fibroblast-derived DNA, respectively. In contrast, Steinemann et al.^(33)^ found evidence that compound heterozygous mutations were the predominant inactivating mechanisms in children with juvenile myelomonocytic leukemia and NF1. Moreover, Fauth et al.^(34)^ reported two trans alterations in one patient, followed by a report of two unrelated familial cases harboring trans double *NF1* mutations by Paterra et al.^(35)^ all of which presented worse clinical phenotypes.

More than 3,600 distinct pathogenic variants have been reported in the *NF1* gene, with approximately 46% classified as extremely rare or private. In contrast, only 31 variants exhibit a prevalence of ≥0.5% among NF1 patients.^(9)^ While all types of pathogenic variants are found in *NF1*, the most frequently reported are nonsense, frameshift, and splicing variants that cause loss of function^(36)^ as observed in the present study. Most variants are intragenic, with approximately 10% representing whole-gene deletions and their flanking regions.^(5,9)^ To date, only a few genotype-phenotype correlations have been established, partially explaining the clinical variability of NF1,^(2,5)^ mainly in patients with particularly complicated forms, such as large deletions in contiguous gene forms,^(4,5)^ increased pNF and symptomatic spinal neurofibromas,^(37)^ or neurofibromatosis-Noonan phenotype.^(38)^

Patient 1 was the only patient in this series who had a negative molecular investigation. He was first examined at 1 year and 3 months to confirm a pediatrician's clinical suspicion of NF1 based on eight CALMs, but did not meet a second diagnostic criterion. During the medical follow-up, the family was anxious about the diagnosis and ordered a gene panel test, which returned negative results for variants of *NF1* and *SPRED1*. Periodic ophthalmological evaluations revealed normal findings. At 6 years of age, he was first noted to have axillary and inguinal freckling. At 8 years of age, he underwent WGS, which also returned a negative result. The case was reexamined using OGM, yielding the same results. In his last clinical evaluation at the age of 10 years, still under puberty development, he continued to present only with multiple CALMs and LMs, without developing LNs or cNF. Growth and neurologic developments were within normal parameters, but he was recently diagnosed with attention deficit hyperactivity disorder. Since technical coverage was considered adequate in this study and OGM discharged chromosomal rearrangements/recombinations, somatic mosaicism is being considered for patient 1. Nevertheless, the patient remains under clinical follow-up, given that additional features of the disorders may emerge over time, and that future reinvestigation using newer techniques may be warranted.

Three recurrent variants were identified in this study. The c.5902C>T p.(Arg1968*) variant was identified as *de novo* in patients 13 and 22; both presented with a similar, benign phenotype in their third decade of life, characterized by CALMs, LMs, LNs, and a few cNFs, along with a single pNF in Patient 13. Similarly, the c.6855C>A p.(Tyr2285*) variant was found in patients 2-M and 16, who exhibited a typical disease until their fourth decade, though patient 16 was diagnosed with breast cancer at age 38.

The other recurrent variant, c.3826C>T p.Arg1276*, was identified in individuals 7 and 25, who presented with distinct, familial phenotypes. stemming from maternal *de novo* events. Besides CALMs and LMs, all affected family members presented with cNF and pNF. Patient 7 exhibited a large pNF on the buttock and upper left thigh. Patient 7-D, diagnosed at age 3, had an extensive cervical pNF requiring a 80% resection and tracheostomy, with residual disease due to involvement of the left external carotid artery and several nerves (accessory, hypoglossal, sympathetic chain, and great auricular chain. Patient 25, a young adult, with a relatively mild presentation, had a mother who uniquely in this series presented with sphenoid wing dysplasia, optic pathway glioma, and a high number of cNF (Figure 2). Although Paria et al.^(39)^ reported two patients with tibial pseudoarthrosis associated with this variant, which contrasts with the present cases, as neither patient had a documented history of long bone changes. Conversely, Bausch et al.^(40)^ described a 50-year-old individual with the same variant, presenting with multiple neurofibromas and associated pheochromocytoma. These findings reinforce significant inter- and intrafamilial variability between genotypes and phenotypes but suggest that this variant may be associated with a greater predisposition to multiple cutaneous and large plexiform neurofibromas.

Patient 20 presented with a neurofibromatosis-Noonan phenotype and was previously described as patient number 20 by Corso et al.^(41)^ carrying the pathogenic variant c.1A>G p.(Met1Val). This variant was previously reported only in individuals with NF1 and/or tumors,^(26,42,43)^ and was associated with features of Noonan syndrome for the first time.

Multiple disorders in a single patient have been estimated to occur in approximately 2%-7.5% of diagnosed genetic diseases, with a higher frequency in consanguineous families. However, double diagnoses due to two pathogenic variants in autosomal dominant morbid genes, which usually arise as *de novo* events, can also occur.^(44,45)^ Owing to its high prevalence, NF1 has been associated with other genetic conditions, including several reports of monogenic disorders and chromosomal abnormalities,^(5)^ representing not only a diagnostic challenge but also therapeutic dilemmas and prognostic uncertainty.^(46)^ The present study also diagnosed three individuals with NF1 and a second genetic disorder.

For Patient 9 with NF1 and spinocerebellar ataxia type 19, both following autosomal dominant inheritance, a segregation analysis could not be performed because the father died. Her family history was negative for both conditions, and parental age was not advanced; the father was 26 years old and the mother was 22 years old at her conception. Spinocerebellar ataxia 19 is a rare autosomal dominant genetic disorder caused by variants in the *KCND3* gene on chromosome 1p13.2 and is characterized by progressive ataxia and cerebellar atrophy. Phenotypic variability exists from a milder late-onset and slowly progressive pure ataxic clinical presentation to a severe early onset form with cognitive impairment, dystonia, and other neurological symptoms. However, the correlations between specific *KCND3* variants and phenotypic outcomes are complex, and no clear genotype-phenotype correlation has been established.^(47)^ Patient 9 fit the classical milder phenotype and seems to represent the first report of spinocerebellar ataxia 19 in Brazil.

Patient 30 presented with NF1 and primary ciliary dyskinesia type 3, the former following autosomal dominant inheritance and the latter with autosomal recessive inheritance. However, segregation analysis was not possible because his father passed away. Nonetheless, family history was negative for both conditions, suggesting that NF1 most likely occurred *de novo*.

The *DNAH5* gene, located on chromosome 5p15.2, is the primary driver of primary ciliary dyskinesia, accounting for 15-29% of such cases in North America and Europe, and 18% in China. Notably, approximately 50% of patients are diagnosed with Kartagener syndrome, a specific subtype characterized by a triad of chronic sinusitis, bronchiectasis, and situs inversus, as observed in Patient 30. Although a precise genotype-phenotype correlation for *DNAH5* remains elusive, the nature and location of variants *DNAH5* may explain the clinical heterogeneity. For example, non-truncated variants, such as those in this patient, appear associated with a less severe presentation than truncated variants, which often lead to earlier disease onset and worse lung function.^(48)^

For these two individuals with additional molecular findings, despite carrying variants classified as VUS, double diagnoses were clinically confirmed in both cases through deep phenotyping. None of these symptoms are features of NF1, supporting a coincidental association between the two diagnoses, rather than a biologically related condition.

The third patient, patient 2-B, had a variant in the *NF1* gene inherited from his father (in addition to a variant of the *TMEM127* gene inherited from his mother) and a 47,XYY/45,X chromosomal constitution. It was not surprising that Patient 2-B presented with NF1 and mosaic double Y syndrome, which is also a frequent condition in the general population. An association between NF1 and XYY has already been reported^(46,49)^ including a triple diagnosis of NF1, XYY, and achondroplasia. ^(50)^

Neurofibromatosis type 1 patients are at a significantly increased risk of developing certain types of cancer. Approximately 10% of plexiform neurofibromas progress to malignancy and the resulting MPNSTs are highly metastatic and incurable.^(4)^ Other frequent tumors in patients with NF1 include astrocytomas, pheochromocytomas, juvenile myelomonocytic leukemia, and breast cancer in women aged <50 years.^(1,3,4)^ Two patients developed breast cancer during the study period. Patient 9 was diagnosed with unilateral invasive ductal carcinoma at the age of 51 years, and patient 16 was recently diagnosed at the age of 38 years with an invasive carcinoma positive for estrogen and progesterone receptors. Other malignancies were reported individually in this series, and except for astrocytoma in patient 26 and MPNST in patients 10-M and 18, other histological types were considered less frequent in NF1. Basal cell carcinoma is a common tumor in the general population, and its occurrence in Patient 11-M likely coincides with the diagnosis of NF1. None of the individuals in this cohort developed hematological malignancies.

Two patients had secondary or incidental findings, both of which were associated with an increased risk of malignancy. Incidental findings have been observed during genomic testing in both clinical and research protocols. In a systematic literature review of incidental findings in whole-genome/exome-sequencing studies, Elfatih et al.^(51)^ found frequencies ranging from 0.59% to 17%, with high variability explained by different study design groups, a lack of consistency in the list of genes, variant nomenclature, filtering criteria, and cohort characteristics. A more reasonable frequency of up to 6% has been reported in another systematic review.^(52)^ The rate of 6.5% observed in the present study was higher than the overall frequency of 3.6% found among the 5,316 individuals investigated in the BRGP. ^(53)^

Family 5 presented an association of pathogenic variants in *NF1* and *BRCA1*, a situation previously described by Ceccaroni et al.^(54)^ which is most likely a case of haplotype segregation, as both genes are located on the long arm of chromosome 17 within approximately 20 cM. Patient 5-M had cholangiocarcinoma at the age of 33 years, which recurred at the age of 48 years and died at the age of 51 years, while patient 5 is being monitored for pancreatic and prostatic tumors.

In Family 2, the mother and brother carried a pathogenic variant of the *TMEM127*, implicated in an increased risk of developing pheochromocytoma. None of the patients presented with this condition, but they were regularly monitored with urinary or plasma-free metanephrine screening.

Finally, the advantages of this study include the assessment of patients by a group of physicians from a single center, which allows for a more homogeneous evaluation and an overall perspective from clinical geneticists. Additionally, we used WGS, the most comprehensive genomic technique currently available, and conducted further investigations using OGM in cases that were initially negative. The main limitations were the sample size and the use of convenience sampling, which included both adult and pediatric individuals. Thus, age-dependent features such as tumor development might have been missing during the study period.

## CONCLUSION

This study presents the largest Brazilian cohort of neurofibromatosis type 1 patients investigated using whole-genome sequencing, identifying three novel germline *NF1* variants and two previously unreported dual molecular diagnoses associated with spinocerebellar ataxia type 19 and primary ciliary dyskinesia type 3. While the diagnostic criteria for neurofibromatosis type 1 are well-established, our whole-genome sequencing findings provide novel insights for genotype-phenotype correlations, future therapeutic strategies, and the management of patients with dual diagnoses.

## Data Availability

The data supporting the findings of this study are available from the corresponding author upon reasonable request.

## References

[1] Legius E, Messiaen L, Wolkenstein P, Pancza P, Avery RA, Berman Y, Blakeley J, Babovic-Vuksanovic D, Cunha KS, Ferner R, Fisher MJ, Friedman JM, Gutmann DH, Kehrer-Sawatzki H, Korf BR, Mautner VF, Peltonen S, Rauen KA, Riccardi V, Schorry E, Stemmer-Rachamimov A, Stevenson DA, Tadini G, Ullrich NJ, Viskochil D, Wimmer K, Yohay K, Huson SM, Evans DG, Plotkin SR, International Consensus Group on Neurofibromatosis Diagnostic Criteria (I-NF-DC) (2021). Revised diagnostic criteria for neurofibromatosis type 1 and Legius syndrome: an international consensus recommendation. Genet Med.

[2] Alesi V, Lepri FR, Dentici ML, Genovese S, Sallicandro E, Bejo K (2022). Intragenic inversions in NF1 gene as pathogenic mechanism in neurofibromatosis type 1. Eur J Hum Genet.

[3] Darrigo LG, Ferraz VE, Cormedi MC, Araujo LH, Magalhães MP, Carneiro RC (2022). Epidemiological profile and clinical characteristics of 491 Brazilian patients with neurofibromatosis type 1. Brain Behav.

[4] McClatchey AI (2007). Neurofibromatosis. Annu Rev Pathol.

[5] Peduto C, Zanobio M, Nigro V, Perrotta S, Piluso G, Santoro C (2023). Neurofibromatosis Type 1: Pediatric Aspects and Review of Genotype-Phenotype Correlations. Cancers (Basel).

[6] Scala M, Schiavetti I, Madia F, Chelleri C, Piccolo G, Accogli A (2021). Genotype-Phenotype Correlations in Neurofibromatosis Type 1: A Single-Center Cohort Study. Cancers (Basel).

[7] Ruggieri M, Praticò AD, Caltabiano R, Polizzi A (2018). Early history of the different forms of neurofibromatosis from ancient Egypt to the British Empire and beyond: first descriptions, medical curiosities, misconceptions, landmarks, and the persons behind the syndromes. Am J Med Genet A.

[8] Ho WY, Farrelly E, Stevenson DA (2022). Evaluation of the impact of the 2021 revised Neurofibromatosis type 1 diagnostic criteria on time to diagnosis. Am J Med Genet A.

[9] Kehrer-Sawatzki H, Cooper DN (2022). Challenges in the diagnosis of neurofibromatosis type 1 (NF1) in young children facilitated by means of revised diagnostic criteria including genetic testing for pathogenic NF1 gene variants. Hum Genet.

[10] Angelova-Toshkina D, Holzapfel J, Huber S, Schimmel M, Wieczorek D, Gnekow AK (2022). Neurofibromatosis type 1: A comparison of the 1997 NIH and the 2021 revised diagnostic criteria in 75 children and adolescents. Genet Med.

[11] Coelho AV, Mascaro-Cordeiro B, Lucon DR, Nóbrega MS, Reis RS, de Alexandre RB (2022). The Brazilian Rare Genomes Project: validation of whole genome sequencing for rare diseases diagnosis. Front Mol Biosci.

[12] Richards S, Aziz N, Bale S, Bick D, Das S, Gastier-Foster J, Grody WW, Hegde M, Lyon E, Spector E, Voelkerding K, Rehm HL, ACMG Laboratory Quality Assurance Committee (2015). Standards and guidelines for the interpretation of sequence variants: a joint consensus recommendation of the American College of Medical Genetics and Genomics and the Association for Molecular Pathology. Genet Med.

[13] Riggs ER, Andersen EF, Cherry AM, Kantarci S, Kearney H, Patel A (2020). Technical standards for the interpretation and reporting of constitutional copy-number variants: a joint consensus recommendation of the American College of Medical Genetics and Genomics (ACMG) and the Clinical Genome Resource (ClinGen). Genet Med.

[14] Quaio CR, Ceroni JR, Pereira MA, Teixeira AC, Yamada RY, Cintra VP (2023). The hospital Israelita Albert Einstein standards for constitutional sequence variants classification: version 2023. Hum Genomics.

[15] Kluwe L, Friedrich RE, Korf B, Fahsold R, Mautner VF (2002). NF1 mutations in neurofibromatosis 1 patients with plexiform neurofibromas. Hum Mutat.

[16] Kayes LM, Riccardi VM, Burke W, Bennett RL, Stephens K (1992). Large de novo DNA deletion in a patient with sporadic neurofibromatosis 1, mental retardation, and dysmorphism. J Med Genet.

[17] Upadhyaya M, Osborn MJ, Maynard J, Kim MR, Tamanoi F, Cooper DN (1997). Mutational and functional analysis of the neurofibromatosis type 1 (NF1) gene. Hum Genet.

[18] Heim RA, Kam-Morgan LN, Binnie CG, Corns DD, Cayouette MC, Farber RA (1995). Distribution of 13 truncating mutations in the neurofibromatosis 1 gene. Hum Mol Genet.

[19] Wimmer K, Schamschula E, Wernstedt A, Traunfellner P, Amberger A, Zschocke J (2020). AG-exclusion zone revisited: lessons to learn from 91 intronic NF1 3′ splice site mutations outside the canonical AG-dinucleotides. Hum Mutat.

[20] Fahsold R, Hoffmeyer S, Mischung C, Gille C, Ehlers C, Kücükceylan N (2000). Minor lesion mutational spectrum of the entire NF1 gene does not explain its high mutability but points to a functional domain upstream of the GAP-related domain. Am J Hum Genet.

[21] Toliat MR, Erdogan F, Gewies A, Fahsold R, Buske A, Tinschert S (2000). Analysis of the NF1 gene by temperature gradient gel electrophoresis reveals a high incidence of mutations in exon 4b. Electrophoresis.

[22] Messiaen LM, Callens T, Mortier G, Beysen D, Vandenbroucke I, Van Roy N (2000). Exhaustive mutation analysis of the NF1 gene allows identification of 95% of mutations and reveals a high frequency of unusual splicing defects. Hum Mutat.

[23] Cawthon RM, Weiss R, Xu GF, Viskochil D, Culver M, Stevens J (1990). A major segment of the neurofibromatosis type 1 gene: cDNA sequence, genomic structure, and point mutations. Cell.

[24] Ainsworth P, Rodenhiser D, Stuart A, Jung J (1994). Characterization of an intron 31 splice junction mutation in the neurofibromatosis type 1 (NF1) gene. Hum Mol Genet.

[25] Wu R, López-Correa C, Rutkowski JL, Baumbach LL, Glover TW, Legius E (1999). Germline mutations in NF1 patients with malignancies. Genes Chromosomes Cancer.

[26] Ko JM, Sohn YB, Jeong SY, Kim HJ, Messiaen LM (2013). Mutation spectrum of NF1 and clinical characteristics in 78 Korean patients with neurofibromatosis type 1. Pediatr Neurol.

[27] Ars E, Serra E, García J, Kruyer H, Gaona A, Lázaro C (2000). Mutations affecting mRNA splicing are the most common molecular defects in patients with neurofibromatosis type 1. Hum Mol Genet.

[28] Nemethova M, Bolcekova A, Ilencikova D, Durovcikova D, Hlinkova K, Hlavata A (2013). Thirty-nine novel neurofibromatosis 1 (NF1) gene mutations identified in Slovak patients. Ann Hum Genet.

[29] Nunes K, Araújo Castro E, Silva M, Rodrigues MR, Lemes RB, Pezo-Valderrama P, Kimura L (2025). Admixture's impact on Brazilian population evolution and health. Science.

[30] Bicudo NP, Germano CM, de Moraes RT, de Avó LR, Ferner RE, Melo DG (2024). Association of sociodemographic and clinical factors with the quality of life of Brazilian individuals with Neurofibromatosis type 1: a cross-sectional study. An Bras Dermatol.

[31] van Minkelen R, van Bever Y, Kromosoeto JN, Withagen-Hermans CJ, Nieuwlaat A, Halley DJ (2014). A clinical and genetic overview of 18 years neurofibromatosis type 1 molecular diagnostics in the Netherlands. Clin Genet.

[32] Alghamdi M, Monies D, Alsohime F, Temsah H, Almodaihsh F, Aldawasri M (2021). Implications of mosaicism in variant interpretation: A case of a de novo homozygous NF1 variant. Eur J Med Genet.

[33] Steinemann D, Arning L, Praulich I, Stuhrmann M, Hasle H, Stary J (2010). Mitotic recombination and compound-heterozygous mutations are predominant NF1-inactivating mechanisms in children with juvenile myelomonocytic leukemia and neurofibromatosis type 1. Haematologica.

[34] Fauth C, Kehrer-Sawatzki H, Zatkova A, Machherndl-Spandl S, Messiaen L, Amann G (2009). Two sporadic spinal neurofibromatosis patients with malignant peripheral nerve sheath tumour. Eur J Med Genet.

[35] Paterra R, Bettinaglio P, Borghi A, Mangano E, Tritto V, Cesaretti C (2022). A Translational Approach to Spinal Neurofibromatosis: Clinical and Molecular Insights from a Wide Italian Cohort. Cancers (Basel).

[36] Gjorgjievska M, Bozhinovski G, Sukarova-Angelovska E, Kocova M, Kanzoska LM, Plaseska-Karanfilska D (2023). Mutational Spectrum and Genotype-phenotype Correlations in Neurofibromatosis Type 1 Patients from North Macedonia: Identification of Ten Novel *NF1* Pathogenic Variants. Balkan Med J.

[37] Koczkowska M, Chen Y, Callens T, Gomes A, Sharp A, Johnson S (2018). Genotype-Phenotype Correlation in NF1: Evidence for a More Severe Phenotype Associated with Missense Mutations Affecting NF1 Codons 844-848. Am J Hum Genet.

[38] Koczkowska M, Callens T, Chen Y, Gomes A, Hicks AD, Sharp A (2020). Clinical spectrum of individuals with pathogenic NF1 missense variants affecting p.Met1149, p.Arg1276, and p.Lys1423: genotype-phenotype study in neurofibromatosis type 1. Hum Mutat.

[39] Paria N, Cho TJ, Choi IH, Kamiya N, Kayembe K, Mao R (2014). Neurofibromin deficiency-associated transcriptional dysregulation suggests a novel therapy for tibial pseudoarthrosis in NF1. J Bone Miner Res.

[40] Bausch B, Borozdin W, Mautner VF, Hoffmann MM, Boehm D, Robledo M, Cascon A, Harenberg T, Schiavi F, Pawlu C, Peczkowska M, Letizia C, Calvieri S, Arnaldi G, Klingenberg-Noftz RD, Reisch N, Fassina A, Brunaud L, Walter MA, Mannelli M, MacGregor G, Palazzo FF, Barontini M, Walz MK, Kremens B, Brabant G, Pfäffle R, Koschker AC, Lohoefner F, Mohaupt M, Gimm O, Jarzab B, McWhinney SR, Opocher G, Januszewicz A, Kohlhase J, Eng C, Neumann HP, European-American Phaeochromocytoma Registry Study Group (2007). Germline NF1 mutational spectra and loss-of-heterozygosity analyses in patients with pheochromocytoma and neurofibromatosis type 1. J Clin Endocrinol Metab.

[41] Corso BM, Simões LO, de Oliveira KM, dos Santos AM, Angeloni LL, de Oliveira RP (2025). Genotype-Phenotype Analysis and New Clinical Findings in a Series of 24 Patients Presenting with Noonan Syndrome and Related Disorders. Mol Syndromol.

[42] Sabbagh A, Pasmant E, Imbard A, Luscan A, Soares M, Blanché H (2013). NF1 molecular characterization and neurofibromatosis type I genotype-phenotype correlation: the French experience. Hum Mutat.

[43] Tsipi M, Poulou M, Fylaktou I, Kosma K, Tsoutsou E, Pons MR (2018). Phenotypic expression of a spectrum of Neurofibromatosis Type 1 (NF1) mutations identified through NGS and MLPA. J Neurol Sci.

[44] Posey JE, Harel T, Liu P, Rosenfeld JA, James RA, Coban Akdemir ZH (2017). Resolution of Disease Phenotypes Resulting from Multilocus Genomic Variation. N Engl J Med.

[45] Rosina E, Pezzani L, Pezzoli L, Marchetti D, Bellini M, Pilotta A (2022). Atypical, Composite, or Blended Phenotypes: How Different Molecular Mechanisms Could Associate in Double-Diagnosed Patients. Genes (Basel).

[46] Muthusamy K, El-Jabali A, Ongie LJ, Dhamija R, Babovic-Vuksanovic D (2022). Neurofibromatosis 1 in the setting of dual diagnosis: diagnostic and management conundrums. Am J Med Genet A.

[47] Avila-Jaque D, Martin F, Bustamante ML, Luna Álvarez M, Fernández JM, Dávila Ortiz de Montellano DJ (2024). The Phenotypic Spectrum of Spinocerebellar Ataxia Type 19 in a Series of Latin American Patients. Cerebellum.

[48] Dong M, Shi X, Zhou Y, Duan J, He L, Song X (2025). Genetic spectrum and genotype-phenotype correlations in DNAH5-mutated primary ciliary dyskinesia: a systematic review. Orphanet J Rare Dis.

[49] Tonouchi E, Morita KI, Harazono Y, Hoshino K, Yoda T (2024). NF1 with 47,XYY mosaicism diagnosed by mandibular neurofibromas. Hum Genome Var.

[50] Pulst SM, Pribyl T, Barker DF, Riccardi VM, Ren M, Yaari H (1991). Molecular analysis of a patient with neurofibromatosis 1 and achondroplasia. Am J Med Genet.

[51] Elfatih A, Mohammed I, Abdelrahman D, Mifsud B (2021). Frequency and management of medically actionable incidental findings from genome and exome sequencing data: a systematic review. Physiol Genomics.

[52] Mitchell LA, Jivani K, Young MA, Jacobs C, Willis AM (2024). Systematic review of the uptake and outcomes from returning secondary findings to adult participants in research genomic testing. J Genet Couns.

[53] Perrone E, Virmond L, Coelho AV, De França M, Moreno CA, Prota JR (2025). ACMG secondary findings in the Brazilian rare genomes project: insights from 5402 genome sequencing. J Hum Genet.

[54] Ceccaroni M, Genuardi M, Legge F, Lucci-Cordisco E, Carrara S, D’Amico F (2002). BRCA1-related malignancies in a family presenting with von Recklinghausen's disease. Gynecol Oncol.

