# Supplementary material for: Whole-genome sequencing in Brazilian patients with neurofibromatosis type 1, including novel variants, incidental findings, and dual diagnoses
Source: Einstein (Sao Paulo). 2026 Jun 23;24:eAO2160. doi: 10.31744/einstein_journal/2026AO2160 (PMC13399297; doi:10.31744/einstein_journal/2026AO2160)
Supplement: Supplementary Material [file 2317-6385-eins-24-eAO2160-Suppl01.pdf]

**SUPPLEMENTARY MATERIAL**

# Whole-genome sequencing in Brazilian patients with neurofibromatosis type 1, including novel variants, incidental findings, and dual diagnoses

Luise Longo Angeloni, Josep Jorente, Ruy Pires de Oliveira Sobrinho, Vera Lúcia Gil-da-Silva-Lopes, Carolina Gama Vidoti-Nascimento, The Brazilian Rare Genomes Project Consortium, Mara Sanches Guaragna, Tarsis Paiva Vieira, Carlos Eduardo Steiner

**DOI: 10.31744/einstein_journal/2026AO2160**

**Table 1S.** Sex, age at first appointment for genetic evaluation, family history, and clinical findings of the 50 individuals with NF1 included in the present study

| Family/patient | | FH | Sex | Age | CALMs | LMs | cNF | pNF | LNs | OPG | SWD | CPT | Other |
|---|---|---|---|---|---|---|---|---|---|---|---|---|---|
| 1 | Proband | - | M | 1y | + | + | - | - | - | - | - | - | ADHD |
| 2 | Proband | + | F | 1y11m | + | + | - | - | + | - | - | - | |
| | Brother | | M | 10y | + | + | - | + | + | - | - | - | Mild ID; 47,XXY/45,X[44:6]; pathogenic variant in *TMEM127* |
| | Mother | | F | 32y | + | + | + | - | - | - | - | - | Pathogenic variant in *TMEM127* |
| | Father | | M | 31y | + | + | + | + | + | - | - | - | |
| 3 | Proband | - | M | 11y | + | + | + | + | + | - | - | - | Dysembryoplastic neuroepithelial tumor at 19y |
| 4 | Proband | - | F | 19y | + | + | + | - | + | - | - | - | LD, mild ID, congenital hip dislocation |
| 5 | Proband | + | M | 9y | + | + | + | - | + | - | - | + | Pathogenic variant in *BRCA1* |
| | Mother | | F | 30y | + | + | + | + | + | - | - | - | Pathogenic variant in *BRCA1*, cholangiocarcinoma at 33y and 47y; died at 51y |
| 6 | Proband | ? | F | 40y | + | + | + | - | ? | - | - | - | |
| 7 | Proband | + | F | 30y | + | + | + | + | + | - | - | - | |
| | Son | | M | 12y | + | + | + | - | + | - | - | - | |
| | Daughter | | F | 4y | + | + | - | + | + | + | - | - | |
| 8 | Proband | - | F | 2y | + | + | - | - | + | - | - | - | |
| 9 | Proband | - | F | 47y | + | + | + | + | + | - | - | - | Breast cancer (unilateral invasive ductal carcinoma) at 51y Cerebellar ataxia at 47y (variant in *KCND3*). |
| 10 | Proband | + | F | 24y | + | + | + | - | + | - | - | - | |
| | Mother | | F | 46y | + | + | + | - | + | - | - | - | MPNST of the right thigh at 45y |
| | Uncle | | M | 62y | + | + | + | - | + | - | - | - | |
| 11 | Proband | + | M | 18y | + | + | + | - | + | - | - | - | |
| | Mother | | F | 43y | + | + | + | + | + | - | - | - | Basal cell carcinoma at 43y |
| | Brother | | M | 24y | + | + | + | - | + | - | - | - | |
| 12 | Proband | - | M | 13y | + | + | + | + | - | - | - | - | |
| 13 | Proband | - | F | 8y | + | + | + | + | + | - | - | - | |
| 14 | Proband | + | M | 12y | + | + | - | - | - | - | - | - | |
| | Mother | | F | 48y | + | + | - | - | ? | - | - | - | |
| | Sister 1 | | F | 22y | + | + | + | - | ? | - | - | - | |
| | Sister 2 | | F | 20y | + | + | - | - | ? | - | - | - | |
| | Brother | | M | 17y | + | + | - | - | ? | - | - | - | |

continue...

...Continuation

**Table 1S.** Sex, age at first appointment for genetic evaluation, family history, and clinical findings of the 50 individuals with NF1 included in the present study

| Family/patient | | FH | Sex | Age | CALMs | LMs | cNF | pNF | LNs | OPG | SWD | CPT | Other |
|---|---|---|---|---|---|---|---|---|---|---|---|---|---|
| 15 | Proband | - | M | 9m | + | + | - | - | + | - | - | - | |
| 16 | Proband | - | F | 31y | + | + | + | + | - | - | - | - | Breast cancer at the age of 38y |
| 17 | Proband | - | F | 21y | + | + | + | + | + | - | - | - | |
| 18 | Proband | - | M | 50y | + | + | + | + | + | - | - | - | Mild ID, MPNST of the dorsum at 51y, died at 53y |
| 19 | Proband | + | F | 2y | + | + | - | - | + | - | - | - | |
| | Uncle | | M | 18y | + | + | + | + | + | - | - | - | |
| | Cousin | | F | 1y | + | - | - | - | - | - | - | - | |
| 20 | Proband | - | M | 15y | + | + | - | - | - | - | - | - | Neurofibromatosis-Noonan phenotype |
| 21 | Proband | - | F | 16y | + | + | - | - | - | - | - | - | |
| 22 | Proband | - | F | 4y | + | + | + | - | + | - | - | - | |
| 23 | Proband | - | F | 4y | + | + | + | + | + | - | - | - | |
| 24 | Proband | - | M | 37y | + | + | + | + | + | - | - | - | |
| 25 | Proband | + | M | 15y | + | + | + | - | - | - | - | - | |
| | Mother | | F | 51y | + | + | + | + | + | + | + | - | |
| 26 | Proband | + | F | 30y | + | + | + | - | + | - | - | - | Pilocytic astrocytoma of the left posterior fossa at 29y |
| | Mother | + | F | 59y | + | + | + | + | + | - | - | - | |
| 27 | Proband | + | M | 2y | + | - | - | - | - | - | - | - | DD, macrocrania |
| | Mother | | F | 32y | + | + | - | - | + | - | - | - | LD, macrocrania |
| | Sister | | F | 5y | + | - | - | - | ? | - | - | - | |
| 28 | Proband | - | F | 29y | + | + | + | + | - | + | - | - | |
| 29 | Proband | + | F | 3y10m | + | + | - | - | - | - | - | + | DD |
| 30 | Proband | - | M | 11m | + | + | - | - | - | - | - | - | *Situs inversus totalis*, recurrent pulmonary infections (variants in *DNAH5*) |

+: present; -: absent; ?: uncertain or unknown; ADHD: attention deficit hyperactivity disorder; cNF: cutaneous neurofibromas; CPT: congenital pseudoarthrosis of the tibia; DD: developmental delay; F: female; FH: Family history; CALM: café-au-lait macules; ID: intellectual deficiency; LD: learning disability; LMs: lentiginous macules; LNs: Lisch nodules; m: months; M: male; MPNST: malignant peripheral nerve sheath tumor; OPG: optic pathway glioma; pNF: plexiform neurofibroma(s); SWD: sphenoid wing dysplasia; y: years.
